# Stabilization of SIRT7 deacetylase by viral oncoprotein HBx leads to inhibition of growth restrictive *RPS7* gene and facilitates cellular transformation

**DOI:** 10.1038/srep14806

**Published:** 2015-10-07

**Authors:** Vijaya Pandey, Vijay Kumar

**Affiliations:** 1 Virology Group, International Centre for Genetic Engineering and Biotechnology, Aruna Asaf Ali Marg, New Delhi-110067, India

## Abstract

Sirtuin-7 (SIRT7) deacetylase exhibits a high selectivity for acetylated H3K18 and has been implicated in the maintenance of malignant phenotype. However, it remains unclear if SIRT7 and H3K18ac play a role in the tumorigenic program driven by oncogenic viruses. We show that ectopically expressed HBx oncoprotein of hepatitis B virus promoted intracellular stability of SIRT7 by salvaging it from ubiquitin-mediated proteasomal degradation. HBx-dependent accumulation of SIRT7 favored H3K18 deacetylation and down-regulated the small ribosomal protein gene, *RPS7*, involved in cell death and DNA damage response. HBx facilitated the recruitment of SIRT7 to *RPS7* promoter thus impeding H3K18ac occupancy and hindering *RPS7* transcription. The antagonistic relationship between SIRT7 and RPS7 was also observed in the HBx transgenic mice, where elevated levels of SIRT7 protein were coincident with low levels of H3K18ac and RPS7. Strikingly, inhibition of cellular deubiquitinase activity restored *RPS7* gene transcription. Further, depletion of endogenous SIRT7 led to decreased cell viability and transformation. The biological relevance of *RPS7* suppression by HBx-SIRT7 axis was evident from ectopic expression of RPS7 which attenuated clonogenicity of cells. Thus, our findings suggest that SIRT7 is a critical regulator of HBx-driven oncogenic program, through its antagonistic impact on growth restrictive ribosomal protein RPS7.

Ribosomal proteins (RPs) have attracted a great deal of attention lately, owed to their extraribosomal functions, in addition to their basic roles in protein bio-synthesis. Till date fourteen RPs have been associated with extra-ribosomal activities majorly related to interception of the well-established p53-Mdm2 axis, thus impinging critically on maintenance of genomic stability and related disorders^1^. Not surprisingly, RPs have been linked with cell proliferation control and their de-regulation with malignancies^2^. Ribosomal protein S7 (RPS7) has recently been documented to interact with Mdm2, leading to stabilization of p53 and modulation of its transactivation function^3^,^4^,^5^,^6^. Besides, the RPS7-Mdm2 interaction has also been involved in stabilization of stress-responding protein GADD45-α^7^. Given its role in DNA damage and p53 stabilization, it is not surprising that RPS7 suppresses ovarian tumorigenesis and metastasis via growth signaling pathways^8^. Collectively these studies imply major roles for RPS7 in sensing DNA damage and cellular stress and averting genomic instability.

More recently, *RPS7* has been reported as one among the select subset of target genes transcriptionally repressed by SIRT7 deacetylase^9^. SIRT7 is a mammalian sirtuin which possesses a highly selective NAD^+^-dependent H3K18ac deacetylase activity and selectively targets genes associated with maintenance of cancer phenotype and tumor formation, as also testified by its elevated expression in several human cancers^9^,^10^,^11^. Besides, it has been shown that SIRT7 is involved in the development and progression of human colorectal cancer (CRC) and thus may serve as a novel prognostic marker and therapeutic target in CRC^12^.

Enrichment of H3K18ac signature at gene promoters is positively correlated with transcriptional activation^13^, while its depletion is associated with aggressive cancer phenotypes and poor clinical outcome^14^,^15^. Interestingly, H3K18ac is also involved in transformation-related epigenetic reprogramming in primary human cells by some viral oncoproteins^16^,^17^,^18^. More specifically, adenoviral E1A oncoprotein and SV 40 large T antigen induce global hypoacetylation of H3K18ac. Incidentally, both adenovirus and SV40 are DNA tumor viruses, which cause H3K18ac depletion, plausibly through mobilization of de-acetylase SIRT7, which perhaps is a general feature of the transformation programs driven by DNA tumor viruses. This event may further affect transcriptional status of a subset of genes such as *RPS7,* having implications in tumorigenesis.

The DNA tumor virus hepatitis B virus (HBV) encodes a viral oncoprotein HBx, which has been established as the major etiological factor associated with HBV-induced human hepatocellular carcinoma (HCC)^19^. HBx enforces its tumorigenic influence in multifarious ways including modulation of host factors involved in cellular signal transduction pathways, transcription, cell cycle, DNA repair, apoptosis and genomic integrity. Interestingly, SIRT7 levels are found to be elevated in a large cohort of HCC patients^20^. The same study also identified SIRT7 as a transcriptional repressor of p21^WAF1/Cip1^ and a target of tumor suppressor micro-RNAs, attesting to its oncogenic potential in hepatocarcinogenesis. However, the prospect of a direct link between HBx and SIRT7 remains elusive.

In the current study we have attempted to address the possibility of manipulation of SIRT7 control and function by viral HBx, to mitigate downstream *RPS7* gene activity and the ramifications of this effect on regulation of cellular transformation ability, in a physiological setting of HBx. Our study elucidates the impact of HBx micro-environment on SIRT7 and H3K18ac cellular status and its bearing on the anti-proliferative role of ribosomal protein S7 in the context of HBV-related HCC.

## Results

### Post-transcriptional up-regulation of SIRT7 levels by viral HBx

**Figure 1:**
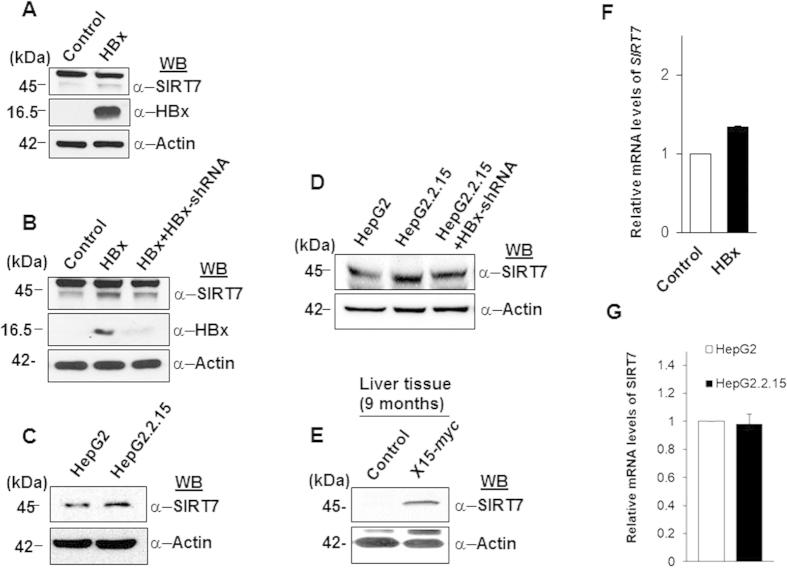
Impact of viral HBx on SIRT7 expression in cell culture and *in vivo*. (**A**,**B**) IHH cells transfected with either empty vector (control) or HBx expression construct (HBx), with or without shRNA directed against HBx expression (HBx+HBx-shRNA) were harvested 48 h later. Whole cell lysates were subjected to western blotting to detect SIRT7, HBx and actin. (**C**,**D**) Western blot analysis of whole cell lysates extracted from either untransfected HepG2 and HepG2.2.15 cells (**C**) or HepG2.2.15 cells transfected with either empty pSILENCER vector or shRNA directed against HBx expression (**D**) followed by immuno-detection of SIRT7 and actin. (**E**) Liver tissue lysates of 9-month old control and X15-*myc* transgenic mice were western blotted for detection of SIRT7 and actin. (**F**) IHH cells were transiently transfected with control or HBx expression construct for 48 h followed by isolation of total RNA. *SIRT7* and *actin* mRNA levels were measured by quantitative reverse-transcriptase PCR (qRT-PCR) using specific primers (Supplemental Table S1). (**G**) *SIRT7* and *actin* mRNA levels were measured as above in total RNA isolated from HepG2 and HepG2.2.15 cells. For western blots belonging to the same experiment, bands pertaining to different proteins were cropped either from the same blot or multiple gels were run under similar experimental conditions. Data (bar diagrams) in (**F**,**G**) are shown as mean ± S.D. of three independent observations.

SIRT7 expression has been suggested to be up-regulated in several cancers and more recently in a large cohort of HCC patients, attributed to down-regulation of specific miRNAs targeting SIRT7 expression in HCC^20^,^21^. We therefore investigated the dynamics of SIRT7 expression in a microenvironment of HBx. IHH cells transiently transfected with either control, HBx alone or with HBx-specific shRNA were examined for the expression of SIRT7 protein. In line with previous reports, we observed a marked increase in the levels of SIRT7 protein in presence of HBx, when compared to control (Fig. 1A,B). Further, on knockdown of HBx expression by specific shRNA, the effect was reversed (Fig. 1B). Complementing these observations, when compared to HBV-negative HepG2 cells, SIRT7 levels were up-regulated in HepG2.2.15 cells (Fig. 1C,D) which harbor chromosomally integrated sequences of HBV genome^22^. Mirroring the effects observed in transfected cell line, knockdown of HBx in HepG2.2.15 cells led to reduction in SIRT7 protein levels (Fig. 1D). Importantly, in agreement with our observations made in transfected IHH cells, the accumulation of SIRT7 protein was also observed in the liver tissues of X15-*myc* transgenic mouse model of HBx-induced HCC (Fig. 1E). Using immuno-histochemistry, we also determined SIRT7 expression profile in the HCC animal model. Further strengthening our observations, as shown in Fig. S1, liver tissues isolated from 20–28 weeks age group (with development of full blown HCC tumor nodules) exhibited intense SIRT7 staining when compared to healthy animals of the same age group. However, we did not observe any correlation of SIRT7 expression in the liver with HCC stage (data not shown).

Since HBx oncoprotein has been implicated in the transcriptional up-regulation of a number of genes^23^,^24^ we reasoned that accumulation of SIRT7 protein levels in HBx-overexpressing cells might be an upshot of transcriptional activation of *SIRT7* gene. To this end, we compared the relative levels of *SIRT7* transcript in IHH cells overexpressing either control or HBx. Figure 1F shows that there was no significant alteration in the relative levels of *SIRT7* mRNA in presence of HBx. In line with this, relative *SIRT7* mRNA levels remained more or less unchanged in HepG2.2.15 cells when compared to HepG2 (Fig. 1G). Thus viral HBx appeared to induce accumulation of SIRT7 protein, most likely through a non-transcriptional mechanism.

### HBx enhances intracellular stability of SIRT7 protein

**Figure 2:**
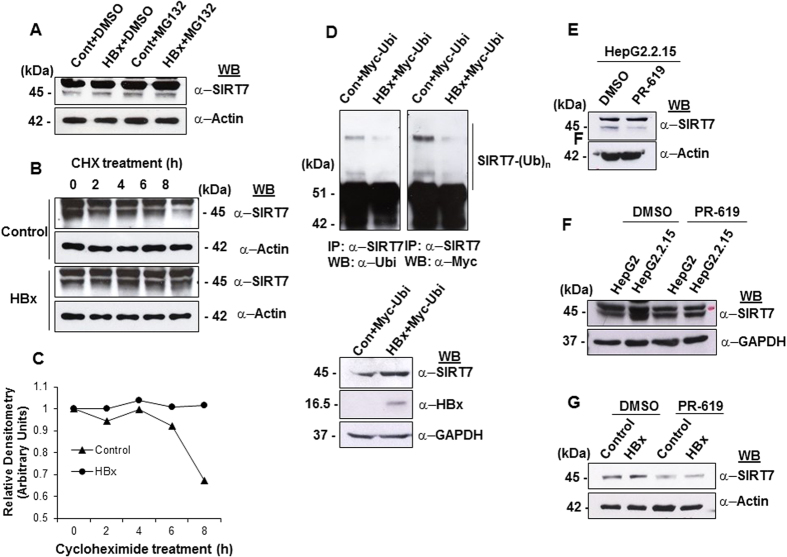
Role of proteasomal degradation and DUB(s) in HBx-mediated stabilization of SIRT7. (**A**) Total cell extracts of IHH cells were transfected with either empty vector (control) or HBx expression construct for 48 h followed by 8 h treatment with 20 μM MG132. The samples were western blotted for detection of SIRT7 and actin. (**B**) Cells were transfected as above and after 48 h treated with cycloheximide (CHX) at a concentration of 100 μg/mL for indicated time points. Changes in the endogenous levels of SIRT7 protein were monitored by western blotting. Actin was used as control. (**C**) Line diagrams showing the levels of SIRT7 protein in control and HBx-transfected cells at indicated time points, after normalization with actin. (**D**) IHH cells overexpressing myc-tagged ubiquitin were co-transfected with either empty vector (control) or HBx expression construct and 48 h post transfection cells were subjected to MG132 treatment as above. Whole cell lysates extracted from these cells were immuno-precipitated with anti-SIRT7 antibody and immuno-complexes were probed with anti-myc antibody. Total protein in cell lysates (10%) was used for detecting SIRT7 and HBx. (**E,F**) HepG2 as well as HepG2.2.15 cells were treated with either DMSO or PR-619, small molecule inhibitor of cellular de-ubiquitinase (DUB) activity at a concentration of 10 μM for 12 h. Total cell lysates were immuno-probed for SIRT7 protein levels. (**G**) IHH cells were transiently transfected with either empty vector (control) or HBx expression construct for 36 h. This was followed by treatment with either DMSO or 10 μM concentration of PR-619 for 12 h. Total cell lysates were immuno-probed for SIRT7 protein levels. In western blots, wither actin or GAPDH was used as internal control. For western blots belonging to the same experiment, bands pertaining to different proteins were cropped either from the same blot or multiple gels were run under similar experimental conditions.

Based on the aforementioned data, we anticipated that the positive influence of HBx on SIRT7 expression might result from modulation of SIRT7 degradation process in presence of HBx. Therefore, we investigated the role of proteasome-mediated degradation in HBx-induced SIRT7 stabilization using MG132, a known inhibitor of proteasome machinery. We observed that MG132 mediated inactivation of proteasome led to accumulation of SIRT7 protein irrespective of HBx presence (Fig. 2A), indicating that plausibly HBx may promote SIRT7 stability by intercepting certain strategic point in the degradation pathway. To further test our hypothesis, we determined the intracellular stability of the protein in presence of HBx, using cycloheximide (CHX) which inhibits protein synthesis. Time-dependent CHX treatment of mock and HBx-overexpressing cells revealed that indeed HBx presence in the cellular milieu prolonged the half-life of SIRT7 protein as compared to control (Fig. 2B,C). Next, we addressed the possibility of alteration of ubiquitination status of SIRT7 in presence of HBx. Indeed, in HBx-overexpressing cells, the ubiquitin-conjugated SIRT7 was detected to a very low level when compared to control (Fig. 2D). Therefore our findings argue for a novel role of HBx in augmenting intracellular stabilization of SIRT7 protein.

Protein ubiquitylation is a master controller of protein function, subcellular localization as well as half-life, thus impacting various cellular processes. Interestingly, deubiquitinases (DUBs) reverse the activity of ubiquitin ligases by removing ubiquitin thus stabilizing the substrate proteins^25^. In this regard, a recent work published from our laboratory revealed HBx-mediated accumulation and hence stabilization of downstream targets of a DUB, USP37^26^. Based on this lead, we surmised that enhanced intracellular stability of SIRT7 could be attributed to this activity of HBx. To this end, we first tested the effect of a pan-DUB inhibitor, PR-619 on SIRT7 levels observed in HepG2.2.15 cells vis-a-vis HepG2 cells. As we show in Fig. 2E,F, inhibition of total cellular DUB activity led to destabilization of SIRT7. Further, HBx could no longer maintain elevated levels of SIRT7 in IHH cells as a consequence of DUB inhibition (Fig. 2G). However, expression of USP37 in IHH cells overexpressing HBx failed to further enhance the stability of SIRT7 (data not shown). In all, these observations bring forth a role of cellular DUB(s) in mediating the stability of SIRT7 in presence of HBx.

### HBx interacts and co-localizes with SIRT7

**Figure 3:**
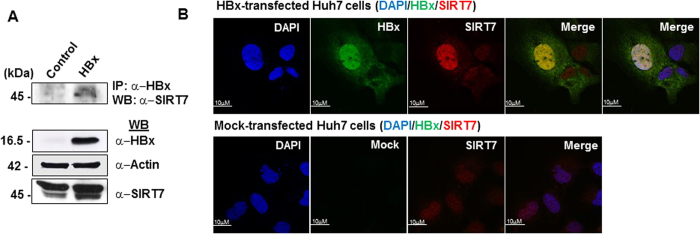
Co-localization of cellular SIRT7 and viral HBx. (**A**) IHH cells transfected with empty vector (control) and HBx expression construct were harvested 48 h later. Whole cell lysates were immuno-precipitated with anti-HBx antibody and immuno-complexes were probed using anti-SIRT7 antibody. Total protein in lysates (10%) was used as input for immuno-detection of HBx and SIRT7. Actin was used as control. (**B**) Huh7 cells were transfected with either empty vector (mock-transfected) or HBx expression construct (HBx-transfected) for 48 h. Cell were fixed with 2% paraformaldehyde followed by immuno-staining with rabbit anti-SIRT7 and mouse anti-HBx as primary antibodies. Secondary antibodies used were Alexa594-conjugated anti-rabbit antibody for SIRT7 (red) and Alexa488-conjugated anti-mouse antibody for HBx (green). Nuclei were stained blue with DAPI. For confocal images, scale bar = 10 μM. For western blots belonging to the same experiment, bands pertaining to different proteins were cropped either from the same blot or multiple gels were run under similar experimental conditions.

While HBx does not possess a DNA binding ability, direct physical interaction with a variety of cellular proteins has been widely demonstrated^27^. These reports, together with our observations that HBx confers stability to SIRT7, prompted us to test the possibility of interaction between SIRT7 and HBx. Our immunoprecipitation analysis revealed SIRT7 in the immunoprecipitates of HBx (Fig. 3A), indicating that SIRT7 and HBx may interact in a cellular milieu. We next sought to determine the subcellular localization of the two interacting partners. For this purpose, Huh7 (Fig. 3B) and U2OS (Fig. S2) cells were transiently transfected with either control or HBx expression constructs, followed by immunofluorescence assay. HBx and SIRT7 expression was confirmed (green and red panels in Figure 3B). Figure 3B and Fig. S2 clearly show that in both Huh7 and U2OS cells, HBx and SIRT7 co-localized primarily in nuclei, although cytoplasmic co-localization was also discernible, especially in U2OS cells (Fig. S2). Interestingly, cells overexpressing HBx, when compared with mock transfected cells, displayed more intense staining for SIRT7, emboldening our previous observation that SIRT7 accumulates in the presence of HBx.

### HBx-dependent accumulation of SIRT7 leads to depletion of H3K18ac signature

**Figure 4:**
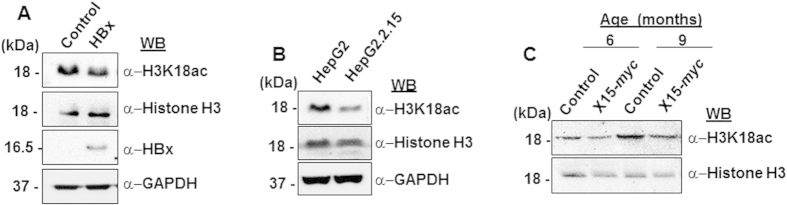
Effect of SIRT7 stabilization on H3K18ac levels in HBx-microenvironment. (**A**) Cell lysates extracted from IHH cells transfected with either empty vector (control) or HBx expression construct, were western blotted for detection of H3K18ac and HBx levels. Histone H3 and GAPDH were used as controls. (**B**) Whole cell lysates obtained from HepG2 and HepG2.2.15 cells were immuno-probed for endogenous levels of H3K18ac. Histone 3 and GAPDH were used as controls. (**C**) Liver tissue lysates of 6 and 9-month old control and X15-*myc* transgenic mice were western blotted for detection of H3K18ac. Histone H3 was used as control. For western blots belonging to the same experiment, bands pertaining to different proteins were cropped either from the same blot or multiple gels were run under similar experimental conditions.

SIRT7 has been reported to possess deacetylase activity with high selectivity for H3K18ac epigenetic mark, low levels of which are strongly associated with cellular transformation programs of viral oncoproteins^9^,^14^. Hence it stands to reason that accumulation of SIRT7 protein in a setting of viral HBx overexpression may bring about global down-regulation of H3K18ac levels. Indeed, we observed diminished levels of H3K18ac in cells overexpressing HBx, when compared to control (Fig. 4A). Consistently, HepG2.2.15 cell line containing chromosomally integrated HBV genomic sequences, exhibited repressed levels of H3K18ac, as compared to control HepG2 cells (Fig. 4B). Further strengthening our observations, H3K18ac levels were depleted in the X15-*myc* transgenic mouse model of HCC (Fig. 4C). Together, these findings suggest SIRT7-mediated negative regulation of global acetylation of H3K18 chromatin mark in a micro-environment of HBx.

### SIRT7 trans-represses *RPS7* gene in the presence of HBx

**Figure 5:**
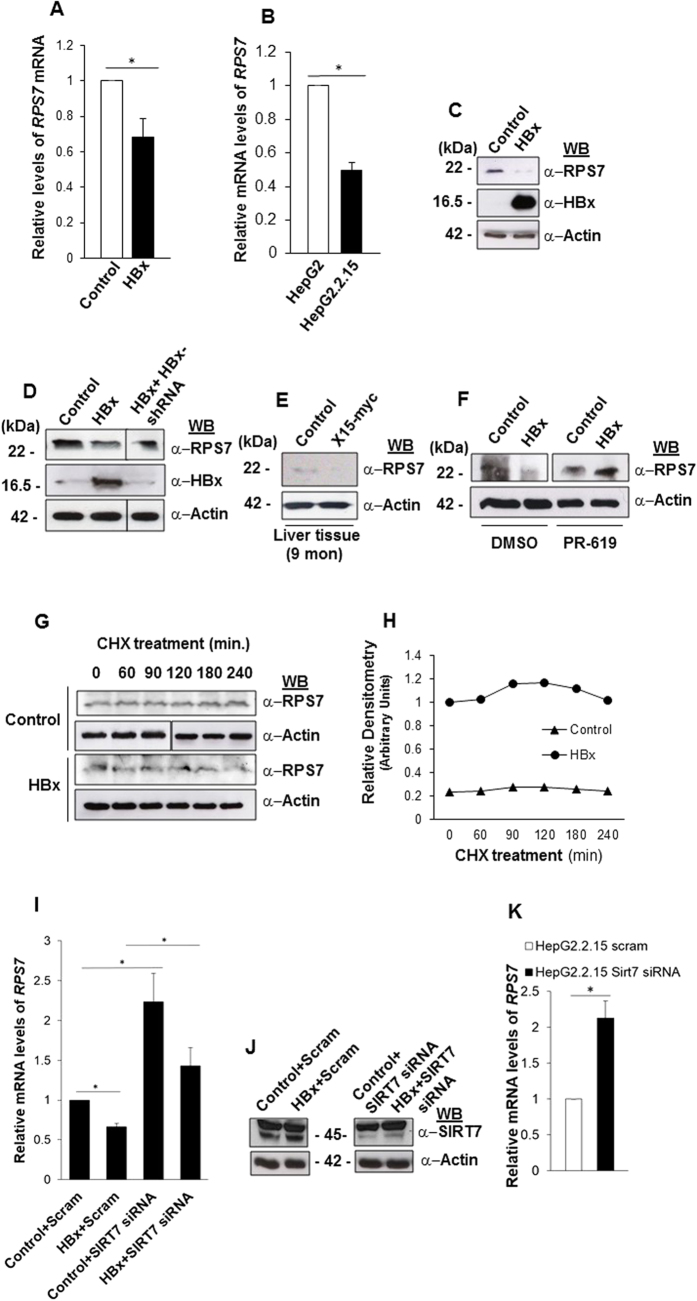
SIRT7-mediated regulation of *RPS7* in presence of HBx. (**A**) IHH cells were transiently transfected with vector (control) and HBx expression construct for 48 h followed by total RNA isolation. *RPS7* and *actin* mRNA levels were measured by qRT-PCR using specific primers (Supplemental Table S1). (B) *RPS7* and *actin* mRNA levels were measured as above using total RNA isolated from HepG2 and HepG2.2.15 cells. (**C**) Lysates extracted from IHH cells transfected as in A, were western blotted for RPS7 and HBx detection. (**D**) Whole cell lysates of cell transfected as in A as well as with HBx shRNA were immuno-blotted to detect RPS7, HBx and actin. (**E**) Liver tissue lysates of 9-month old control and X15-*myc* transgenic mice were western blotted to detect RPS7. (**F**) IHH cells transfected as in A for 36 h were treated with either DMSO or 10 μM concentration of PR-619 for additional 12 h. Total cell lysates were immuno-probed for RPS7 protein. (**G**) IHH cells were transfected as in A and after 48 h treated with cycloheximide (CHX) at a concentration of 100 μg/mL for indicated time points. Changes in the endogenous RPS7 protein levels were measured using immuno-detection with specific antibody. (**H**) Line diagrams showing levels of RPS7 protein in control and HBx-transfected cells at indicated time points, after normalization with actin. (**I**) IHH cells were transfected with either scrambled (Scram) or SIRT7 specific (SIRT7 siRNA) siRNA. After 24 h, cells were transfected with either vector (control) or HBx expression construct followed by RNA isolation for estimation of *RPS7* and *actin* mRNA levels as in A. (**J**) Total cell extracts from cells treated as in (**I**) were western blotted for immuno-detection of SIRT7 and actin. Actin was used as control in all western blot experiments. (**K**) HepG2 and HepG2.2.15 cells were transfected with either scrambled (Scram) or SIRT7 specific (SIRT7 siRNA) siRNA for 48 h, followed by total RNA isolation. For western blots belonging to the same experiment, bands pertaining to different proteins were cropped either from the same blot or multiple gels were run under similar experimental conditions. *RPS7* and *actin* mRNA levels were measured as described in A. Data (bar diagrams) are shown as mean ± S.D. of three independent observations. # and * indicate statistically significant difference at p < 0.05 and p < 0.01, respectively.

*RPS7* is one among the select subset of genes that have been demonstrated to be transcriptionally repressed by SIRT7-mediated H3K18 hypoacetylation on their promoters in cancer cells^9^. Additionally, a number of RP genes including *RPS7*, have been found to be mis-regulated in several cancers^1^. In light of these reports, we hypothesized that global hypo-acetylation of H3K18 observed in presence of HBx might impinge upon the transcriptional regulation of *RPS7*. To this end, we checked mRNA levels of *RPS7* in HBx overexpressing IHH cells. As shown in Fig. 5A, HBx overexpression in IHH cells, in contrast to control, significantly attenuated the relative mRNA levels of *RPS7*. In addition, HepG2.2.15 cells also displayed a 2-fold decrease in the relative levels of *RPS7*, compared to HepG2 cells (Fig. 5B). Further, RPS7 protein levels in HBx overexpressing cells corroborated well with mRNA profiles (Fig. 5C). Importantly, knockdown of HBx expression using specific shRNA in HBx-overexpressing IHH cells restored the basal levels of RPS7 protein (Fig. 5D). In addition, RPS7 levels were also found to be diminished in liver tissues of transgenic mice (Fig. 5E), mirroring the effects observed in cell lines. Since SIRT7 levels were adversely affected by inhibition of DUB activity in HBx-overexpressing cells, we expected to observe a downstream effect on RPS7 protein levels. Indeed, RPS7 protein levels were restored significantly upon DUB treatment even in the presence of HBx (Fig. 5F). In order to confirm that the down-regulation of RPS7 carried out by SIRT7 is primarily a transcriptional effect, we checked the effect of HBx on half-life of RPS7 protein using cycloheximide treatment. Confirming our hypothesis, there was no appreciable change in the half-life of RPS7 protein in CHX-treated HBx-overexpressing cells when compared to control (Fig. 5G,H). Hence, these findings validate that HBx suppresses *RPS7* via SIRT7 through transcriptional mechanism. In addition, these observations raise the possibility that SIRT7-mediated repression of *RPS7* may be attributed to H3K18 deacetylation activity of SIRT7.

In order to evaluate direct role of SIRT7 in HBx-dependent transcriptional repression of *RPS7*, we depleted cellular levels of SIRT7 in presence of HBx and measured its effect on relative transcript levels of *RPS7*. In line with our previous observations, relative mRNA levels of *RPS7* were restored upon depletion of SIRT7, despite the presence of HBx (Fig. 5I,J). Similar pattern was recorded in SIRT7 siRNA treated HepG2.2.15 cells (Fig. 5K). Therefore, we infer that HBx debilitates the transcriptional status of ribosomal gene *RPS7*, via promotion of the intracellular stability of SIRT7, a trans-repressor of *RPS7*.

### Viral HBx engages cellular DUB activity to stabilize SIRT7 and promote its recruitment on *RPS7* promoter

**Figure 6:**
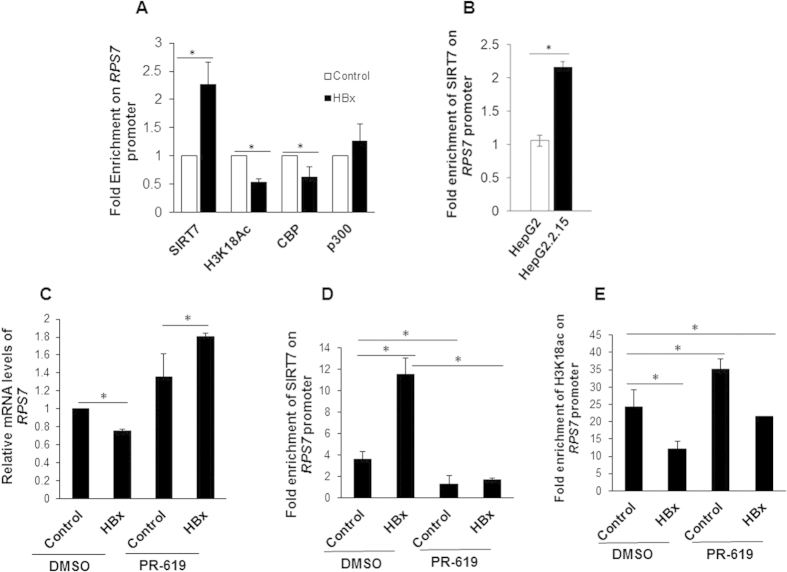
Reciprocal influence of HBx and DUB inhibitor on SIRT7 and H3K18ac occupancy on *RPS7* promoter. (**A**) IHH cells were transfected with either empty vector (control) or HBx expression construct for 48 h. Cells were processed for ChIP assay using antibodies against SIRT7, H3K18ac, CBP and p300. Normal rabbit serum was used as negative control in ChIP assays. Fold enrichment over mock due to occupancy of indicated protein on *RPS7* promoter was measured by ChIP-qPCR using primers specific for *RPS7* promoter (Supplemental Table S1). (**B**) HepG2 and HepG2.2.15 cells were harvested and processed for ChIP assay using anti-SIRT7 antibody as described above. (**C**) Huh7 cells were transiently transfected with either empty vector (control) or HBx expression construct for 36 h followed by treatment with either DMSO or 10 μM PR-619 for 12 h. Total RNA was isolated and used for estimation of *RPS7* and *actin* mRNA levels by qRT-PCR using specific primers (Supplemental Table S1). (**D**,**E**) IHH cells were transfected with either empty vector (control) or HBx expression construct for 36 h followed by treatment with either DMSO or 10 μM PR-619 for 12 h and processed for ChIP assay using antibodies against SIRT7 or H3K18ac. Normal rabbit serum was used as negative control in ChIP assays. Fold enrichment over mock due to occupancy of indicated protein on *RPS7* promoter was measured by ChIP-qPCR using primers specific for *RPS7* promoter (Supplemental Table S1). Data (bar diagrams) are shown as mean ± S.D. of three independent observations. # and * indicate statistically significant difference at p < 0.05 and p < 0.01, respectively.

It has been reported earlier that the recruitment of SIRT7 to the promoters of genes with tumor suppressive activities such as *RPS7* couples H3K18 deacetylation to transcriptional repression^9^. Our observation on the transcriptional down-regulation of *RPS7* in presence of HBx led us to hypothesize that the elevated levels of SIRT7 in presence of HBx might facilitate its recruitment to *RPS7* promoter, thus suppressing its expression. Examination of the recruitment of SIRT7 to *RPS7* promoter, using ChIP- quantitative PCR (ChIP-qPCR) confirmed increased promoter occupancy of SIRT7 in presence of HBx when compared to control (Fig. 6A). Notably, HepG2.2.15 cells also displayed higher levels of SIRT7 bound to *RPS7* promoter, contrary to HepG2 cells (Fig. 6B). Interestingly, we also observed a marked down-regulation of H3K18ac levels coinciding with increased SIRT7 recruitment on *RPS7* promoter upon HBx overexpression (Fig. 6A). Global levels of H3K18ac *in vivo* have been shown to be maintained by activities of p300/CBP histone-acetyltransferases^16^,^17^. Therefore, a decreased H3K18ac mark in HBx microenvironment piqued our curiosity to determine the status of CBP and p300 recruitment to *RPS7* promoter. We observed that while p300 levels remained unchanged, CBP recruitment was adversely affected with a ~2 fold decrease (Fig. 6A). Collectively, aforementioned findings suggest existence of HBx-controlled negative regulatory axis, wherein increased SIRT7 recruitment and CBP detachment from *RPS7* promoter transcriptionally silences the gene mediated by reduction of H3K18ac occupancy.

Given the observation that HBx-mediated SIRT7 accumulation was impaired following inhibition of total cellular DUB activity, we reasoned that it may have a bearing on the transcriptional regulation of *RPS7*. To test this possibility, we measured mRNA levels of *RPS7* in control and HBx-overexpressing cells in absence and presence of DUB inhibitor. As compared to untreated cells overexpressing HBx, those with obliterated DUB activity showed strong induction of *RPS7* evident from ~2-fold increase in relative mRNA levels as measured by qRT-PCR (Fig. 6C). Thus, these findings suggest that inhibition of DUB activity rescues *RPS7* from transcriptional down-regulation brought about by HBx-SIRT7 nexus. The up-regulation of *RPS7* by inhibition of total DUB activity raised the possibility that the mechanism involved may entail decreased recruitment of SIRT7 to *RPS7* promoter. To test this, we asked whether treatment of HBx overexpressing cells with DUB inhibitor can impair the binding of SIRT7 to the promoter of *RPS7*. Indeed, our results showed that increased recruitment of SIRT7 to *RPS7* promoter observed in HBx micro-environment is severely affected when cells were treated with DUB inhibitor (Fig. 6D). Consistent with these observations, inhibition of total DUB activity also led to restoration of acetylated levels of H3K18 on *RPS7* promoter (Fig. 6E). These data support the notion that cellular DUB activity is indispensable for SIRT7 to maintain its inhibitory influence on acetylation status of H3K18 and hence transcriptional state of *RPS7* promoter in the presence of HBx.

### HBx-SIRT7 axis and RPS7 reciprocally regulate cell survival and anchorage-independent cell growth

**Figure 7:**
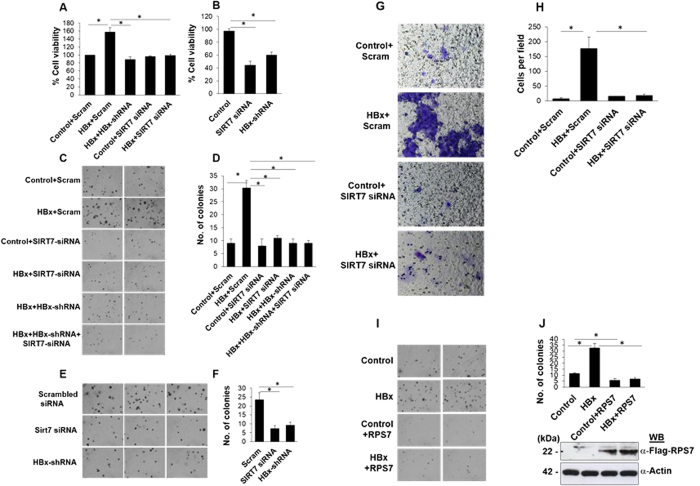
Role of SIRT7 and RPS7 on viability, anchorage independent growth and invasiveness of cells in HBx micro-environment. (**A**) IHH cells, seeded at 0.3 × 10^6^ cells per well in 6-well format, were transfected with either scrambled (Scram) or SIRT7 specific (SIRT7 siRNA) siRNA. After 24 h, cells were transfected again with either empty vector (control), HBx expression construct or HBx along with shRNA directed against its expression (HBx+HBx-shRNA). After 24 h, cell viability was determined using 3-(4, 5-methylthiazol-2-yl)-2,5-diphenyl-tetrazolium bromide (MTT) assay and calculated as percentage normalized with control. (**B**) HepG2.2.15 cells were seeded and transfected as above and cell viability was determined as above. (**C**,**E**) IHH cells were transiently transfected with either scrambled (Scram) or SIRT7 specific (SIRT7 siRNA). At the same time HepG2.2.15 cells were transiently transfected with either scrambled siRNA, SIRT7 specific siRNA or shRNA directed against HBx expression (HBx-shRNA). After 24 h, IHH cells were re-transfected for another 24 h with either empty vector (control), HBx expression construct or HBx along with shRNA directed against its expression (HBx+HBx-shRNA). Forty eight hours after first transfection, IHH and HepG2.2.15 cells were seeded for the soft agar assay. Representative images of microscopic analysis (with a 10X objective) of soft agar assay are shown. (**G**) Huh7 cells were transiently transfected with either scrambled siRNA or SIRT7 specific siRNA for 24 h followed by re-transfection for another 24 h with either empty vector (control) or HBx expression construct. Cells were then trypsinized and seeded on the inner surface of 8 μm insert. Cells were allowed to migrate for 20–24 h following which migrated cells on the outer surface were fixed with paraformaldehyde and stained with crystal violet. Stained cells were observed under light microscope (using 20X objective). Representative images of migrated Huh7 cells have been shown. (**H**) Bar diagram represents average number of cells migrated per field calculated from 5 independent fields on the each insert. The experiment was done in triplicate. (**I**) IHH cells were transiently transfected with vector and HBx expression construct either alone or along with Flag-tagged RPS7 expression vector. After 48 h, cells were treated as in C and E. In addition, total cell extracts were used to detect expression of Flag-tagged RPS7 protein (bottom panel of **J**). (**D**,**F**,**J**) Mean number of foci in 20 fields on each plate obtained on repeating the experiment three times are shown in the bar diagrams. For western blots belonging to the same experiment, bands pertaining to different proteins were cropped either from the same blot or multiple gels were run under similar experimental conditions. Data (bar diagrams) are shown as mean ± S.D. of three independent observations. # and * indicate statistically significant difference at p < 0.05 and p < 0.01, respectively.

The question that arises from the aforementioned findings is whether DUB mediated stabilization of SIRT7 in presence of HBx has a further biological consequence on the cell beyond transcriptional down-regulation of *RPS7* via impairment of H3K18 acetylation on its promoter. Several reports have demonstrated oncogenic potential of SIRT7, implicating its role in properties necessary for carcinogenesis such as anchorage-independent growth and tumor formation in xenograft assays^9^,^20^,^21^. In order to determine whether increased SIRT7 levels induce oncogenic transformation in presence of HBx, we followed several approaches. In the first approach, we measured cell viability using MTT assay in response to SIRT7 depletion in HBx-transfected IHH cells. Figure 7A shows that as opposed to untreated HBx-transfected cells, those depleted of SIRT7 showed lower viability, suggesting that SIRT7 exercises a positive influence on the growth of cells and its depletion promotes cell death even in the presence of viral oncoprotein HBx. Surprisingly, the percent of cells viable upon treatment with HBx shRNA and SIRT7 siRNA was comparable, indicating that the higher viability of HBx transfected cells could be majorly attributed to SIRT7 and that SIRT7 is a critical determinant of HBx-mediated transformation program. As part of the second approach, HepG2.2.15 cells which carry HBV genome in their chromosome, were depleted of SIRT7 and HBx, individually, followed by measurement of cell viability using MTT assay. As shown in Fig. 7B, both HBx and SIRT7 depletion brought about a significant drop in cell viability and consistent with results obtained in IHH cells, the extent of cell death induced by HBx knockdown was at par with that of SIRT7 silencing. In the third approach, we assessed the pro-survival potential of SIRT7 in HBx micro-environment using a soft agar assay. Figure 7C,D show that siRNA-mediated SIRT7 depletion led to a dramatic reduction in the number of colonies formed by HBx overexpressing cells. Reminiscent of cell survival assay, cells lost their colony formation ability comparably upon individual silencing of HBx and SIRT7. In addition, there was only slight further reduction in the number when HBx and SIRT7 were depleted together. In agreement with observations made in IHH cells, soft agar assay performed in HepG2.2.15 cells also revealed that colony forming ability of cells suffered a severe setback upon depletion of HBx and SIRT7 (Fig. 7E,F). Similarly, a migration assay using Transwell invasion method revealed that depletion of SIRT7 in HBx-overexpressing cells attenuated the invasive potential bestowed upon the cells by expression of HBx oncogene (Fig. 7G). The aforementioned cell survival, transformation and migration assays strongly support that SIRT7, acting true to its oncogenic character, directs cellular transformation program in HBx micro-environment.

Based on our observations that expression of RPS7 is strongly repressed by SIRT7 in presence of HBx, it is reasonable to expect that there must be a physiologically relevant function of RPS7 that underlies the need for its ablation in micro-environment of a viral oncoprotein such as HBx. Indeed, RPS7, having tumor suppressive function, is known to modulate MDM2-p53 interaction, with consequences such as cell death and arrest of proliferation of cancer cells^4^. In addition, association of RPS7 with MDM2 has implications in regulation of stress responding proteins engaged in DNA damage response^7^. Hence it is conceivable that re-introduction of RPS7 in HBx setting may prove to be catastrophic to the oncogenic program set in motion by SIRT7. To test this hypothesis, we performed soft agar assay in cells co-transfected with HBx and RPS7 expression constructs. As depicted in Fig. 7G,H, overexpression of RPS7 in presence of HBx led to a severe reduction in the colony formation ability of cells, clearly highlighting that RPS7 acts as a tumor suppressor to restrict the oncogenic potential of HBx-SIRT7 axis.

**Figure 8:**
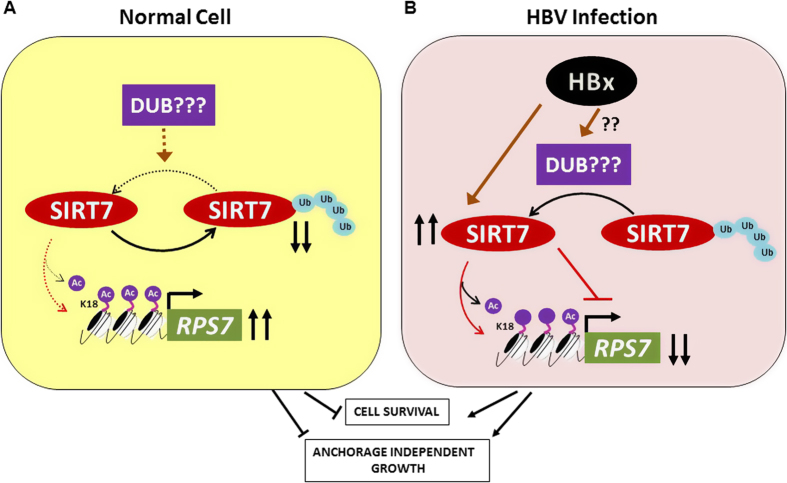
Scheme showing regulation of *RPS7* gene by SIRT7 deacetylase in the presence of HBx. (**A**) In a normal cell, low basal levels of SIRT7 protein ensure *RPS7* promoter occupancy with acetylated H3K18 leading to uninterrupted transcription of tumor suppressor RPS7. (**B**) However, in the presence of viral HBx, there is accumulation of SIRT7 which causes de-acetylation of H3K18 on *RPS7* promoter leading to silencing of the gene and induction of oncogenic features such as enhanced cell viability and anchorage independent growth of cells.

Collectively, as shown in Fig. 8, our findings indicate that viral oncoprotein HBx induces high expression of SIRT7, partly promoted by cellular DUB activity, and instigates repression of *RPS7* through deacetylation of H3K18 occupying *RPS7* promoter, thus reversing its growth restrictive impact.

## Discussion

Along with genetic events, cancer-associated alterations in histone modifications are important determinants in the initiation and progression of tumorigenesis^15^. Recent studies have shown that cellular levels of H3K18ac predict clinical outcome in multiple cancers, with lower levels predicting significantly poorer survival probability^14^. Establishing a strong correlative link between H3K18ac and oncogenic transformation, viral oncoproteins have been reported to induce hypoacetylation of H3K18, causing cellular epigenetic reprogramming^16^,^17^,^18^. Moreover, H3K18ac is a selective substrate of SIRT7, the activity of which is necessary for maintaining oncogenic features of human cancer cells^9^. The present study highlights the influence of viral oncoprotein HBx on the expression of SIRT7 and probes its downstream effects on deacetylation of H3K18 on gene promoter, impinging on cellular transformation program.

Apart from its role in oncogenic transformation, SIRT7 has been demonstrated to be instrumental in maintaining hepatic metabolic homeostasis specifically liver lipid metabolism^28^,^29^. In light of these reports, our observations that HBx, strongly associated with HCC, interacts and co-localizes with SIRT7, along with augmenting its stability, are particularly intriguing. Considering the role of SIRT7 in liver physiology, it’s de-regulation by a viral oncoprotein encoded by HBV favors the idea that it may also possess a crucial function in oncogenic program driven by HBx in liver. Indeed in our study, cancer-promoting attributes such as cell survival, anchorage independent growth and invasiveness, which are supported by HBx, suffered a major setback when cellular levels of SIRT7 were depleted either using RNAi or inhibition of DUB activity, arguing for a strong role of SIRT7 in furthering the oncogenic program set in by HBx. In support of this notion, we observed elevated levels of SIRT7 in the liver tissues of X15-*myc* transgenic mice, not only in total protein extracts but also in tissue sections. Adding further relevance to our findings, SIRT7 levels have also been found to accumulate in liver cancer tissues isolated from a large cohort of HCC patients, where its expression is correlated with tumor grade^20^. Importantly, colorectal cancer patients with high SIRT7 expression exhibit decreased overall and disease-free survival^12^. Our study, taken together with these evidences, proposes that SIRT7 overexpression in cancer including HCC has important clinico-pathological implications, including value of SIRT7 as a prognostic and therapeutic candidate.

It is of interest that relative mRNA levels of *SIRT7* have been shown to be affected in a variety of cancers, in certain cases by miRNA mediated regulation^20^,^30^. Specifically, the 3′ UTR of *SIRT7* mRNA has been shown to possess binding sites for miRNAs such as hsa-miR-125b, miR-125a-5p and miR-125b^20^,^30^. However, in our study although the protein levels of SIRT7 were consistently found to be elevated, relative mRNA levels remained unaltered (both in transiently transfected cells as well as those which stably harbor HBV genome). Thus, we focused majorly on investigation of SIRT7 protein stability and not regulation of *SIRT7* mRNA by miRNAs. We believe the difference in observations made by us and others, may stem from the difference in choice of experimental systems, which in our case was cells overexpressing HBx. Nevertheless, it shall be of great interest to examine the effect of HBx on miRNAs known to participate in regulation of SIRT7 expression, considering that HBx is involved in promoter regulation of several miRNAs^31^. Interestingly, according to a recent development SIRT7 has been shown to engage in epigenetic silencing of miRNA gene, miR-34a^32^. While the mechanism elucidated in this study which involves H3K18 deacetylation mediated epigenetic silencing of miR-34a, is in harmony with our observations, it also widens the spectrum of SIRT7 functions. Apart from this study, Barber *et al.*^9^ identified 35 non-protein coding genes, promoters of which are bound by SIRT7, however, a direct evidence linking SIRT7 to regulation of miRNAs remains unveiled.

Role of de-ubiquitinases is becoming increasingly apparent in cancer with swiftly expanding list of roles in pathways directing cellular transformation^33^. Further, DUBs are manipulated by viral oncoproteins, promoting viral invasion and pathogenesis^26^. However, thus far no evidence of involvement of DUB activity in regulation of SIRT7 stability either under normal conditions or under the influence of a viral oncoprotein, has come to light. In this respect, our study is by far the first to indicate the role of cellular DUB activity in regulation of cellular status of SIRT7 in the context of HBx oncoprotein. It is worth noting that recently a study published from our laboratory^26^ elucidated a positive influence of HBx on the activity of DUB USP37. Thus, we wondered if the effect of DUB inhibitor on SIRT7 protein levels observed in presence of HBx was dependent upon HBx-mediated up-regulation of USP37. However, we did not observe any appreciable changes in SIRT7 levels following the over-expression of USP37. Hence we envisage a scenario where a DUB, other than USP37, participates in conferring stability to SIRT7 and this DUB is under positive influence of HBx, explaining loss of SIRT7 stabilization upon treatment of cells with DUB inhibitor (PR-619). These observations build enough grounds to spark hunt for cellular DUB activities possibly involved in SIRT7 regulation, albeit not being the focal theme of the current study. A close perusal of the DUBs engaged in promoting SIRT7 stability may also prove useful in testing and formulizing an effective combinatorial therapeutic regime employing specific SIRT7 and DUB inhibitors.

Just as high selectivity of H3K18 for SIRT7 deacetylase, its acetylation is also specifically catalyzed by histone acetyltransferases p300 and its closely related paralogue CREB-binding protein (CBP)^16^,^17^. Interestingly, the interaction of adenoviral oncoprotein E1A with p300 and CBP is considered crucial for transformation process^16^. In this vein, HBx too has been previously reported to interact with CBP^34^. Of particular interest, we show that SIRT7 recruitment to *RPS7* promoter in presence of HBx is concomitant with decreased occupancy of CBP. This result along with the observation that HBx and SIRT7 interact in cellular milieu, suggests that HBx favors SIRT7 recruitment to *RPS7* promoter at the expense of CBP, consequently causing hypoacetylation of H3K18. However, at variance with this hypothesis, interaction of HBx with p300/CBP has been associated with increased expression of cell cycle and growth promoting genes, hence is deemed crucial for HBx-induced predisposition of cells to transformation^34^. In light of these paradoxical functions of HBx, it appears that the viral protein enforces either spatial or temporal isolation of its interaction with CBP in order to ensure on one hand CBP-dependent gene expression, while on the other, its dislodgement from *RPS7* promoter.

Role of HBx in perturbing regulation of ribosomal protein genes has been demonstrated previously. In a study published from our laboratory, the liver tissue of X15-*myc* oncomouse exhibited sustained and elevated expression of ribosomal protein gene *RPS27a*, which was associated with HBx-mediated hepatocarcinogenesis^35^. However, the present study suggests a negative influence of HBx on expression of another ribosomal gene *RPS7*, which participates in growth inhibitory functions, as indicated previously and now in this report. RPS7 moonlights in extra-ribosomal capacity to integrate DNA damage stress signaling with p53-Mdm2 axis^36^. Hence it is fair to argue that RPS7 may be a target of choice for oncogenes in order to ensure uninterrupted proliferation. Indeed, RPS7 has been demonstrated to stabilize p53 in lung cancer cells treated with immuno-modulatory anti-tumor protein^6^. Another study reported suppressive role of RPS7 in ovarian tumorigenesis^8^. Interestingly, RPS7 is also a reported target of transcriptional repression by deacetylase SIRT7, with implications in cancerous growth^9^. Keeping in view of anti-proliferative extra-ribosomal properties of RPS7 and it being a transcriptional target of SIRT7, it was imperative for us to assess effect of up-regulated SIRT7 on *RPS7* transcriptional status in presence of HBx. That SIRT7 mediates transcriptional silencing of RPS7 in presence of HBx, attests to growth-restrictive attributes of RPS7, which are also amply exemplified in cell survival and colony formation assay.

Thus, the present study describes SIRT7 deacetylase as a critical protagonist of oncogenic program set in motion by HBx (Fig. 8). The positive impact of HBx on stability of SIRT7 entails lowered ubiquitination of SIRT7 as well as involvement of a cellular DUB activity, deciphered using proteasome and DUB inhibitors. Decreased occupancy of H3K18ac on *RPS7* promoter, commensurate with increased recruitment of SIRT7 leads to transcriptional attenuation of promoter activity in presence of HBx. Mitigation of RPS7, which engages in p53 signaling and DNA damage response, does not augur well for genomic integrity of the cell as it fosters cell survival, anchorage independent growth and invasiveness. Importantly, these tumorigenic consequences are reversed upon either ectopic restoration of RPS7 or depletion of SIRT7 and HBx. These observations also reflect translational value of RPS7 as a potential candidate for therapy involving its restoration or reversal of epigenetically silenced state in tumor tissues overexpressing SIRT7 to counter the oncogenic phenotype. Besides, RPS7 may also be investigated for its diagnostic potential in HBV-associated HCC.

Our observation that viral oncoprotein HBx causes H3K18 hypoacetylation, has not merely added another viral oncoprotein to the list of H3K18ac targeting oncoproteins of viral origin, but also reinforced the paradigm that DNA tumor viruses such as adenovirus, SV40 and now HBV might share similar mechanisms of epigenetic reprogramming for coercing normal cells to transform. It is also important to note that the revelation of H3K18ac as a common target of many viral oncoproteins including HBx, has underscored its yet under-appreciated role in transcriptional regulation of genes and warrants a quest into the physiological function of this histone mark. Considering the wealth of information viruses have offered to molecular biologists over the decades, it is not too far-fetched to expect that activities of viral oncoproteins may harbor important lessons about functions and mechanisms of fundamental epigenetic events in normal as well as cancer cells.

## Methods

### Recombinants, chemicals and reagents

Construction of the eukaryotic expression vectors for wild-type HBx and shRNA against HBx were as described previously^23^,^37^. Full length human *RPS7* gene was amplified and cloned in pCMV-Tag2b vector with N-terminal FLAG tag. Myc-ubiquitin construct was kindly provided by Dr. Michael MC Lai (Institute of Molecular Biology, Academia Sinica, Taipei, Taiwan). Antibodies were obtained from following sources: SIRT7 and H3K18ac from Millipore, HBx, actin, GAPDH, histone H3, ubiquitin, Myc, CBP and p300 antibodies from Santa Cruz Biotechnology, RPS7 antibody from Proteintech and anti-FLAG antibody from Sigma-Aldrich. Seakem-LE agarose used for colony formation assay was obtained from Lonza. Inhibitors PR-619 and MG-132 and MTT used for cell survival assay were purchased from Sigma-Aldrich. Cycloheximide was procured from Calbiochem. Control and SIRT7-specific siRNA were purchased from Invitrogen. For quantitative-PCR, iTaq™ Universal SYBR® Green Supermix from Biorad was used. For immuno-histochemistry, Lab Vision DAB Quanto detection system was procured from Thermo Scientific.

### Cell cultures, DNA and siRNA transfections

Maintenance of immortalized human hepatocytes (IHH), human hepatoma Huh7, HepG2 and HepG2.2.15 cells was carried out in Dulbecco’s modified Eagle’s medium (DMEM from Invitrogen) supplemented with 10% fetal bovine serum (FBS) at 37 °C in a humidified atmosphere with 5% CO2. For DNA transfections, cells were seeded at a density of 0.3 × 10^6^, 0.6 × 10^6^ and 1 × 10^6^ cells respectively for 6-well culture plate, 60-mm or 100-mm culture dishes and subsequently transfected with 1.5, 2.0 and 5 μg of indicated plasmids respectively of each kind of dish using Lipofectamine (Invitrogen) according to the manufacturer’s instructions. For siRNA transfections, cells seeded at a density of 1 × 10^6^ in 100 mm dishes were transfected with 20 nM of either scrambled (control) or SIRT7 specific siRNA using manufacturer’s protocol (Invitrogen) for 24 h.

### Immunoprecipitation and western blotting

Cell lysates, prepared in cell lysis buffer, were used for protein estimation with Bradford’s reagent. For immunoprecipitation, equal amounts of protein diluted in cell lysis buffer were incubated with 1 μg of indicated antibody overnight at 4 °C. The immune complexes were isolated using protein A-Sepharose beads (GE Healthcare). Samples prepared in 2X SDS dye were resolved on SDS-PAGE, electro-transferred to nitrocellulose membrane and protein bands were visualized using enhanced chemiluminescence (ECL, Invitrogen). For western blotting equal amounts of protein, obtained from whole cell lysates, were resolved on SDS-PAGE and processed as described above.

### X15-*myc* transgenic mouse model

Development of X15-myc transgenic mouse model has been described earlier^38^. The design and use of transgenic mouse was approved by Institutional Animal Ethics Committee (approval no. ICGEB/AH/2004/05/VIR-13) and the experimental methods were carried out according to the approved guidelines. The liver tissue samples were surgically removed at the indicated age of the animals and processed for western blotting studies. Expression of SIRT7 was investigated in the liver of these mice by immuno-histochemistry (IHC). Briefly, 2 μm thick paraffin sections of liver on glass slides were dewaxed in xylene, rehydrated in decreasing concentrations of alcohol followed by treatment with 0.3% hydrogen peroxide and Proteinase K-mediated antigen retrieval processing. The tissue sections were then blocked in 3% BSA followed by incubation with either IgG control or anti-SIRT7 and further processing for IHC analysis using Thermo Scientific Lab Vision Quanto Detection system as per the manufacturer’s protocol. The sections were then counterstained with hematoxylin followed by dehydration and mounting with DPX. The sections were analyzed under Nikon Eclipse 80*i* microscope (with 60x objective).

### RNA isolation and real-time quantitative PCR

RNA extraction was carried out using TRIzol reagent (Invitrogen) as per the supplier’s instructions. Reverse transcription PCR (RT-PCR) was performed using M-MuLV reverse transcriptase (Fermentas) according to manufacturer’s guidelines. The real time quantitative PCR (qPCR) was done using specific primer pairs (Supplementary Table 1) and 2X PCR mix as per the manufacturer’s protocol (Biorad).

### Immunofluorescence Microscopy

Huh7 cells were transfected with HBx expression construct for 48 h followed by fixation in PBS with 2% paraformaldehyde for 20 minutes at room temperature, permeabilization with 0.4% Triton X-100 in PBS for 15 minutes at room temperature, and blocking with PBS containing 5% bovine serum albumin (BSA). Immunostaining was performed using mouse anti-HBx and rabbit anti-SIRT7 antibodies. Unbound-antibodies were washed away with PBS followed by incubation with Alexa488-conjugated goat anti-mouse and Alexa594-conjugated goat anti-rabbit antibodies purchased from Invitrogen. The nuclei were stained with DAPI. Slides were observed under 60X magnification of A1R (Nikon).

### Inhibitor treatment

The chemical inhibitors were used at following concentrations for given durations of time: DUB inhibitor PR-619 at 10 μM for 12 h, proteasomal inhibitor MG-132 at 20 μM for 8 h and protein synthesis inhibitor cycloheximide (CHX) at 100 μg/mL for indicated time points.

### *In-vitro* ubiquitination assay

IHH cells were transfected as described above with either control or HBx along with Myc-Ubiquitin constructs for 40 h. Following this, cells were treated with 20 μM MG-132 for 8 h before harvesting and total cell extracts were prepared in cell lysis buffer supplemented with protease inhibitor cocktail. For all samples equal amounts of proteins were incubated with 1 μg of anti-SIRT7 antibody overnight at 4 °C. The immuno-precipitated complexes were resolved on SDS-PAGE followed by immuno-detection of ubiquitin conjugated SIRT7 using anti-ubiquitin antibody.

### Chromatin Immuno-precipitation (ChIP)

ChIP assays were performed as per the manufacturer’s protocol (Upstate Biotechnology). Briefly, 10 × 10^6^ cells were cross-linked with formaldehyde (1%), sonicated at 4 °C (5 pulses for 10 seconds each at 30% amplitude) and centrifuged at 13,000 rpm at 4 °C for 10 minutes to obtain clear lysate. Cell lysates were pre-cleared for 2 h with protein-A–Sepharose beads (Amersham Biosciences) and incubated overnight with indicated antibodies. The immune complexes were pulled down using protein-A–Sepharose beads pre-blocked with BSA and salmon sperm DNA. After multiple washing steps in low salt, high salt and lithium chloride containing buffers, DNA was eluted using elution buffer made in 0.1 M NaHCO_3_ and 1% SDS followed by purification using phenol-chloroform extraction. The eluted DNA was analyzed using real time quantitative PCR (qPCR) as described above and specific primers.

### Cell viability assay

IHH cells, seeded at 0.3 × 10^6^ cells per well in 6-well format, were transfected as described above. Subsequently, cell viability was determined by adding 200 μg/mL of 3-(4, 5-dimethylthiazol-2-yl)2,5-diphenyl-tetrazolium bromide per well followed by incubation at 37 °C for 20 minutes to allow formation of formazan. Cells were then washed with 1X PBS and 200 μL of dimethylsulfoxide (DMSO) was added to dissolve formazan. Absorbance was measured at 570 nm and DMSO was used as reference.

### Colony formation assay

IHH cells and HepG2.2.15 cells seeded in a 6-well format were transfected as described above. Post-transfection, cells were trypsinized, and 3 × 10^5^ cells were mixed with 1 mL of 0.8% SeaPrep agarose in 2X DMEM and layered over 0.75% SeaPrep agarose in 2X DMEM containing 10% FBS. The plates were incubated at 37 °C for 14–21 days and observed regularly for formation of colonies. Bright-field images of transformed colonies were captured with a Nikon ECLIPSE *Ti* inverted microscope (with a 10X objective), and the number of foci formed on each plate was calculated by counting colonies in 20 random fields.

### Transwell invasion assay

The migration assay was performed using Transwell cell culture inserts (Corning) of 8 μm pore size and 6-well plates. Briefly, Huh7 cells were transfected with either 20 nM scrambled or SIRT7 specific siRNA for 24 hours followed by transfection with either control or HBx expression construct for additional 24 hours. Following this, cells were trypsinized and counted under microscope. 8 μm inserts placed in wells of a 6-well plate were pre-incubated in DMEM with 10% FCS for 1 hour at 37^o^C and 5% CO_2_. As per the manufacturer’s protocol, 3 × 10^5^ cells were seeded onto the inner surface of the insert. After incubation for 20–24 hours, media from inside the inserts and wells was aspirated and the non-migrated cells on the inner side of the insert were removed using a moist cotton swab. The cells that had migrated to the outer side of the inserts were fixed in 2% paraformaldehyde and stained with 1% crystal violet for 15 minutes followed by counting under Nikon Eclipse 80*i* microscope (with a 20x objective). Average number of cells was calculated from cells counted in 5 separate fields per insert and the experiment was performed in triplicate.

### Statistical analysis

Data are expressed as means ± s.e.m. The statistical significance of results was calculated using Student’s t-test. P-value of <0.05 and <0.01 was considered to be significant.

## Additional Information

**How to cite this article**: Pandey, V. and Kumar, V. Stabilization of SIRT7 deacetylase by viral oncoprotein HBx leads to inhibition of growth restrictive *RPS7* gene and facilitates cellular transformation. *Sci. Rep.* **5**, 14806; doi: 10.1038/srep14806 (2015).

## Supplementary Material

Supplementary Information

Supplemental data
