# Author Correction: Stabilization of SIRT7 deacetylase by viral oncoprotein HBx leads to inhibition of growth restrictive *RPS7* gene and facilitates cellular transformation

**DOI:** 10.1038/s41598-020-67899-2

**Published:** 2020-06-29

**Authors:** Vijaya Pandey, Vijay Kumar

**Affiliations:** Virology Group, International Centre for Genetic Engineering and Biotechnology, Aruna Asaf Ali Marg, New Delhi, 110067 India

Correction to: *Scientific Reports* 10.1038/srep14806, published online 07 October 2015

This Article contains errors.

As a result of an error during figure assembly, an image for Control second panel in Figure 7I is a duplication of an image for Control + Scram second panel in Figure 7C. The correct Control panel for Figure 7I is shown below as Figure 1.

**Figure 1:**
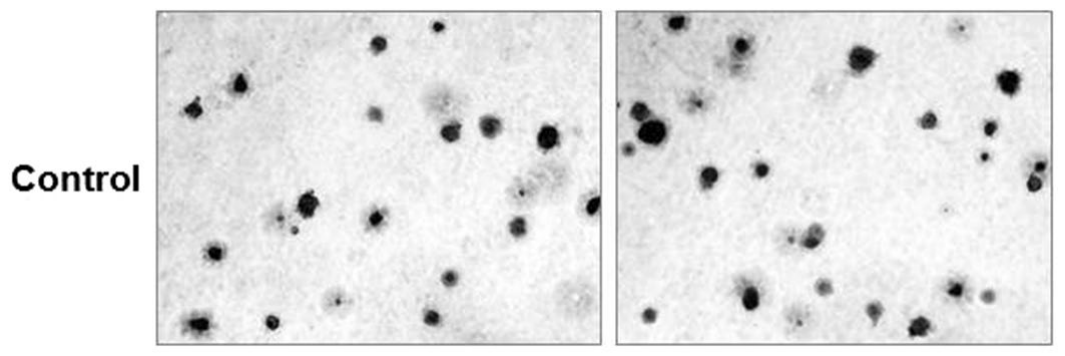
.

Additionally, this affects the graph in Figure 7J which was obtained from data in Figure 7I. Corrected graph for Figure 7J is shown below as Figure 2.

**Figure 2:**
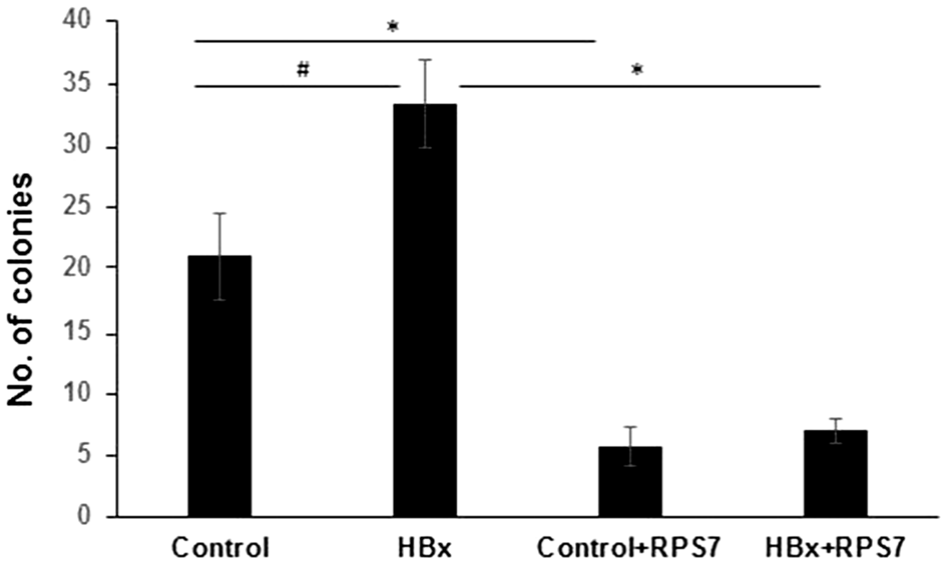
.

These changes do not affect overall conclusions of the Article.

