# Supplementary material for: Stabilization of SIRT7 deacetylase by viral oncoprotein HBx leads to inhibition of growth restrictive RPS7 gene and facilitates cellular transformation
Source: Sci Rep. 2015 Oct 7;5:14806. doi: 10.1038/srep14806 (PMC4595800; doi:10.1038/srep14806)
Supplement: Supplementary Information [file srep14806-s1.pdf]

Stabilization of SIRT7 deacetylase by viral oncoprotein HBx leads to inhibition of growth restrictive *RPS7* gene and facilitates cellular transformation

Vijaya Pandey and Vijay Kumar

Supplementary Information

Raw data related to western blots of different figs.

FIG. 1

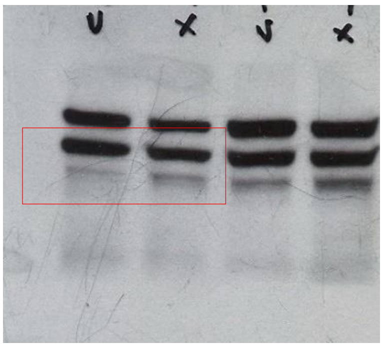

Fig. 1A - SIRT7

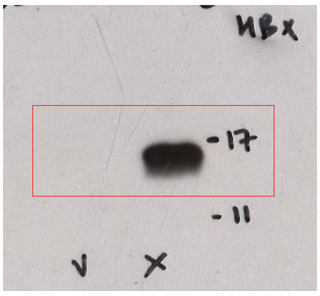

Fig. 1A- HBx

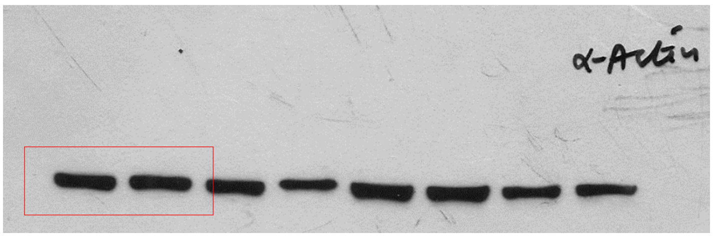

Fig. 1A - Actin

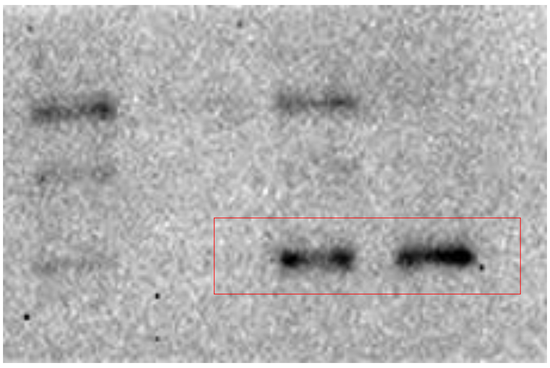

Fig 1C - SIRT7

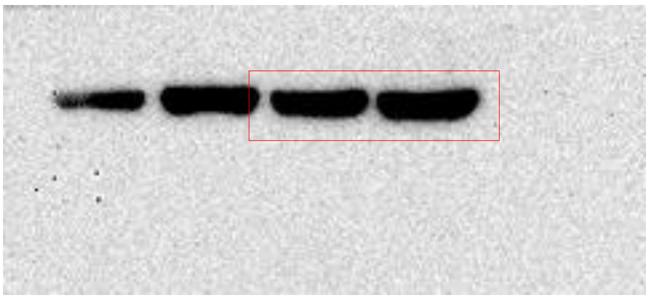

Fig. 1C - Actin

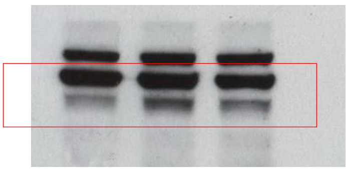

Fig. 1B - SIRT7

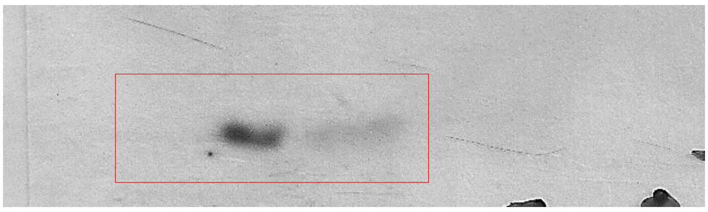

Fig. 1B - HBx

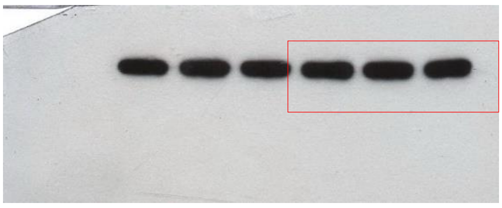

Fig. 1B - Actin

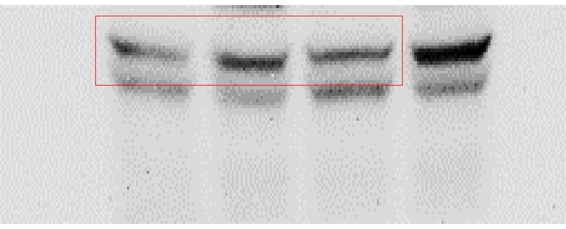

Fig. 1D- SIRT7

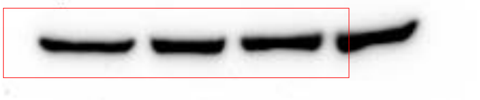

Fig. 1D- Actin

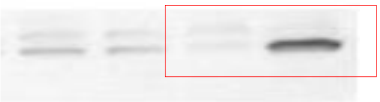

Fig. 1E - SIRT7

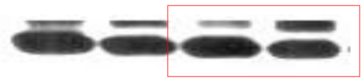

Fig. 1E - Actin

FIG. 2

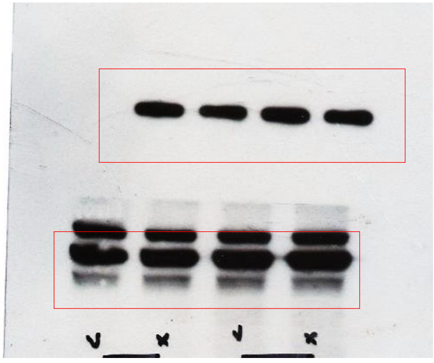

Fig. 2A – Actin
Fig. 2A – SIRT7

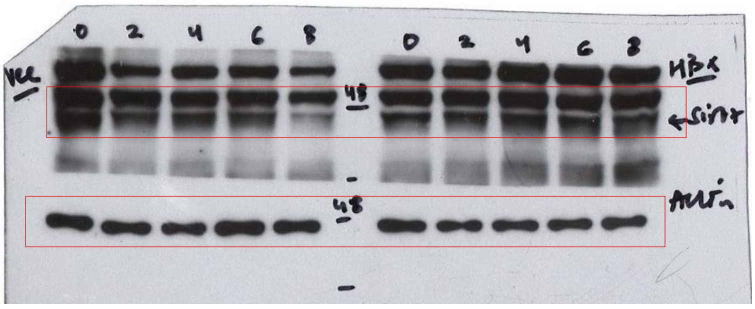

Fig. 2B – SIRT7
Fig. 2B – Actin

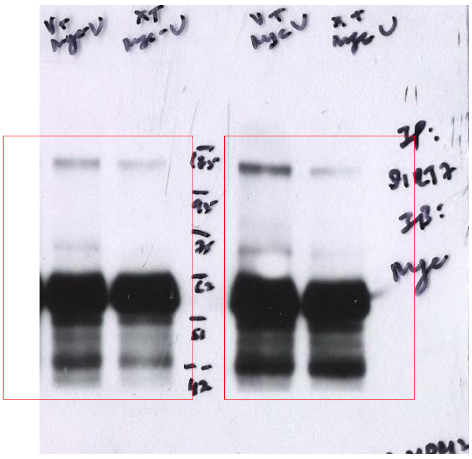

Fig. 2D – SIRT7 Ubi

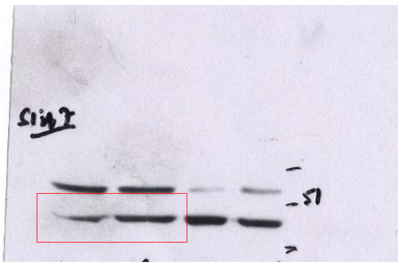

Fig. 2D - SIRT7

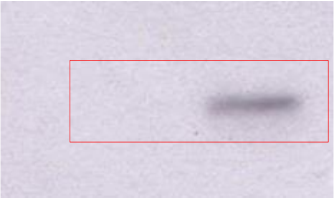

Fig. 2D- HBx

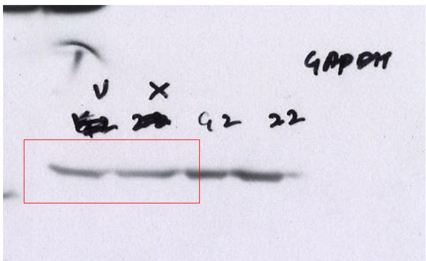

Fig. 2D - GAPDH

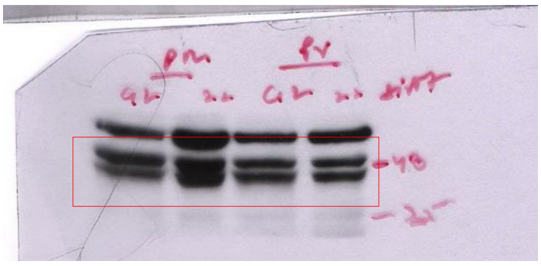

Fig. 2F - SIRT7

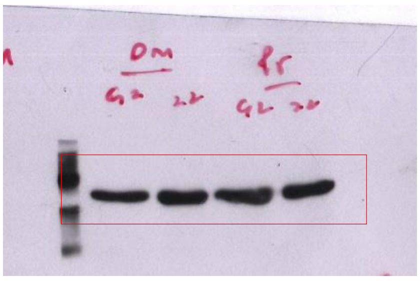

Fig. 2F - Actin

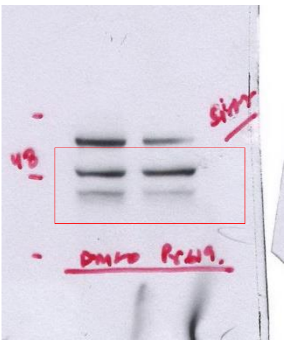

Fig. 2E – SIRT7

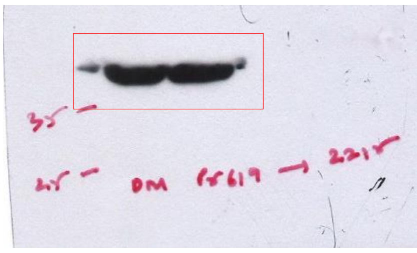

Fig. 2E - Actin

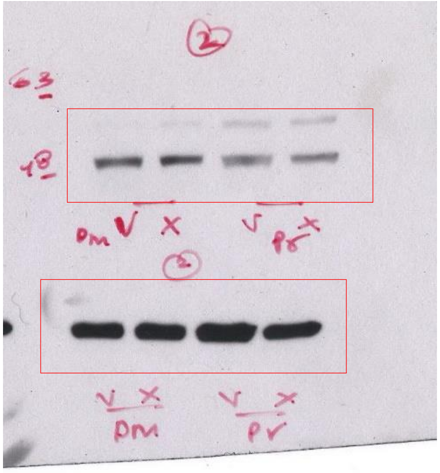

Fig. 2G – SIRT7

FIG. 3

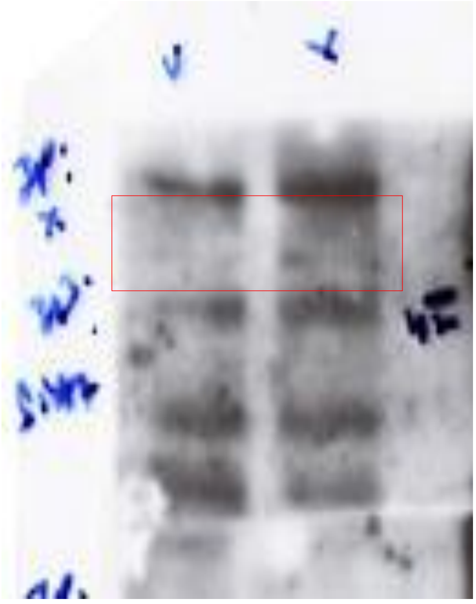

Fig. 3A – IP – HBx; WB – SIRT7

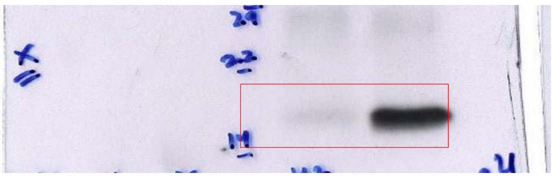

Fig. 3A – WB - HBx

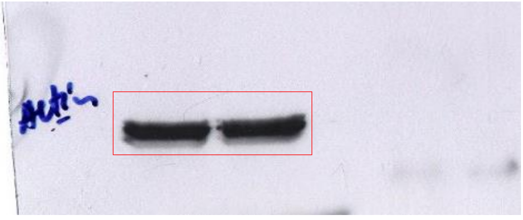

Fig. 3A - Actin

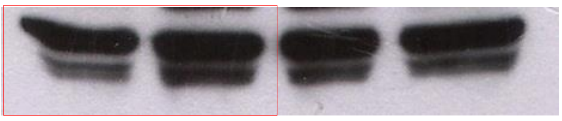

Fig. 3A – SIRT7

FIG. 4

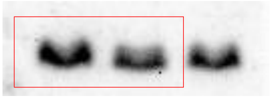

Fig. 4A - H3K18ac

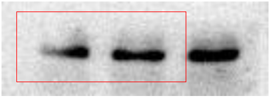

Fig. 4A – Total H3

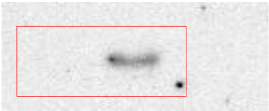

Fig. 4A - HBx

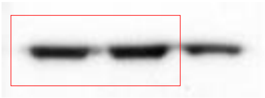

Fig. 4A - GAPDH

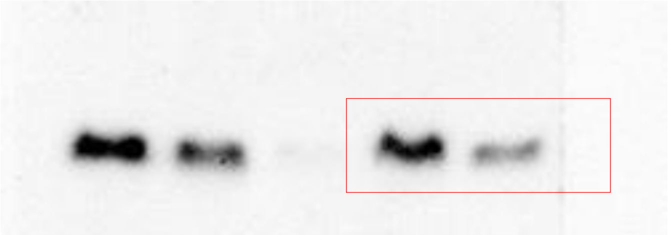

Fig. 4B - H3K18ac

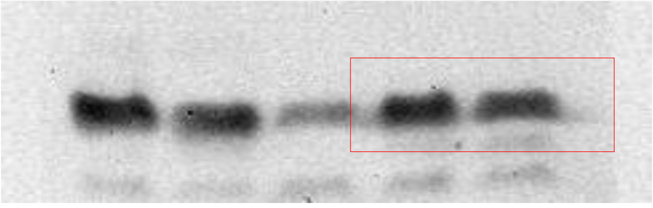

Fig. 4B – Histone H3

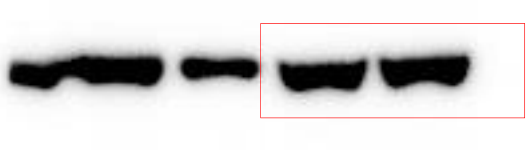

Fig. 4B - GAPDH

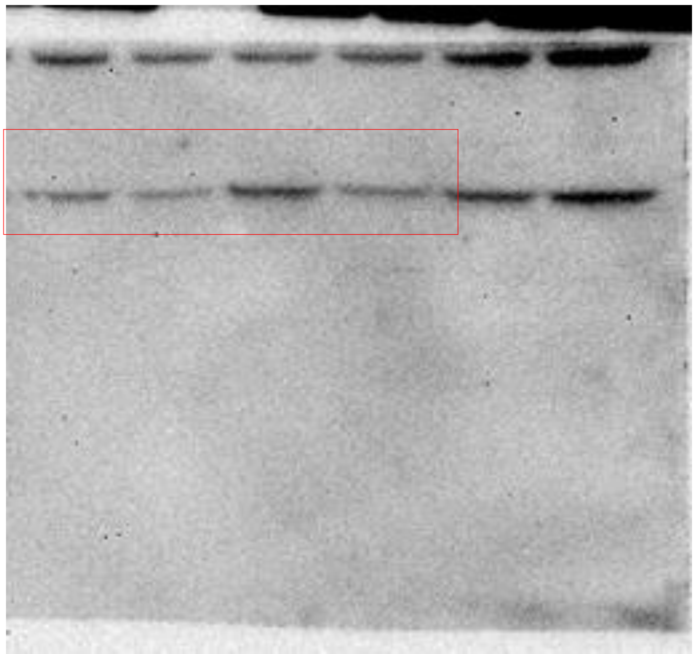

Fig. 4C - H3K18ac

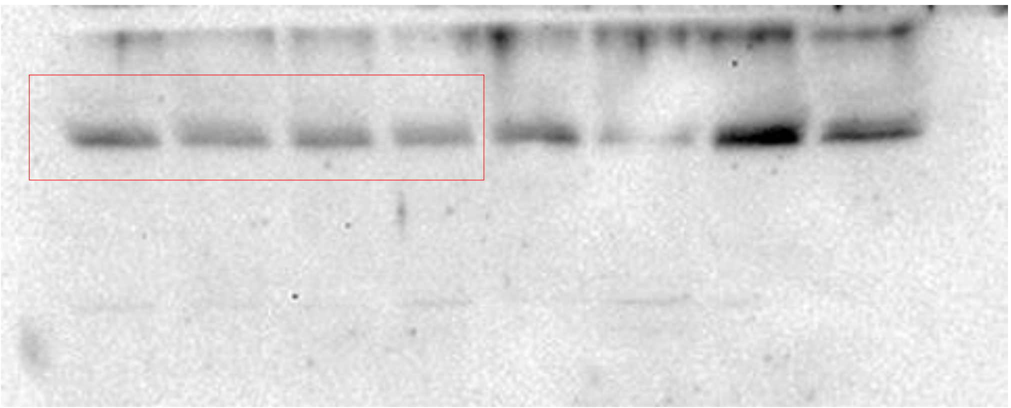

Fig. 4C – Histone H3

FIG. 5

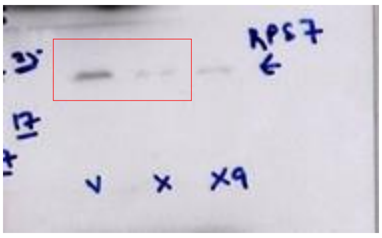

Fig. 5C - RPS7

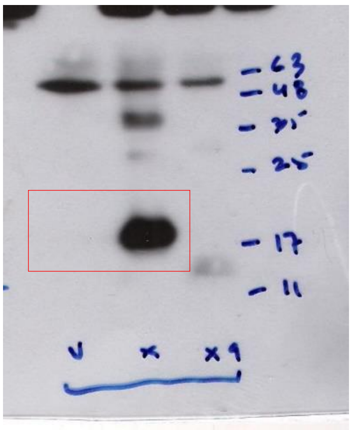

Fig. 5C - HBx

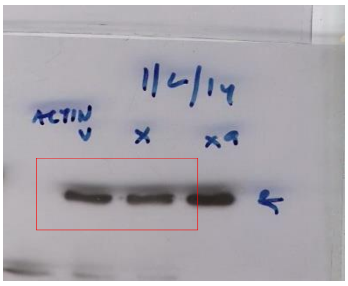

Fig. 5C - Actin

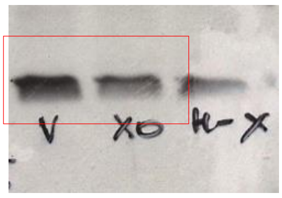

Fig. 5D - RPS7 (Lanes 1 & 2)

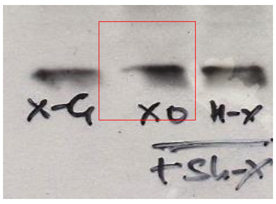

Fig. 5D - RPS7 (Lane 3)

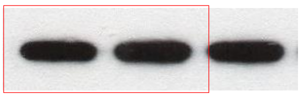

Fig. 5D - RPS7 (Lanes 1 & 2)

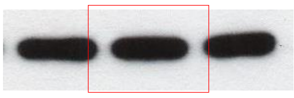

Fig. 5D – Actin (Lane 3)

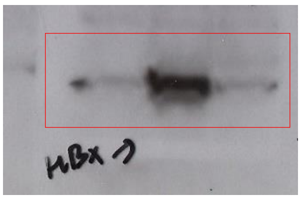

Fig. 5D - HBx

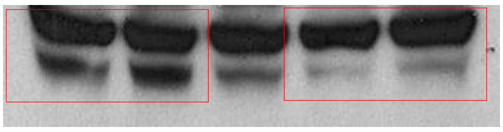

Fig. 5J - SIRT7

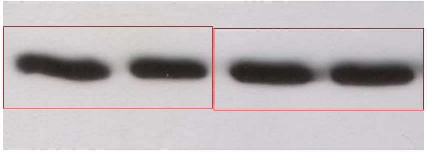

Fig. 5J - Actin

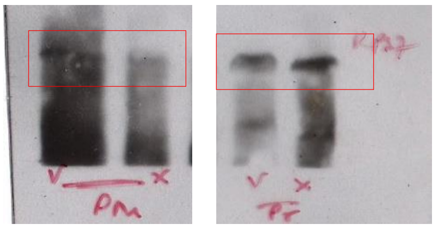

Fig. 5F- RPS7

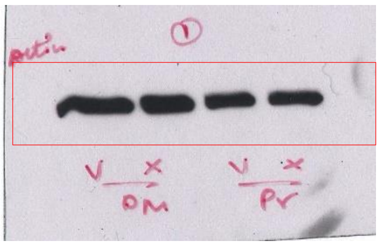

Fig. 5F- Actin

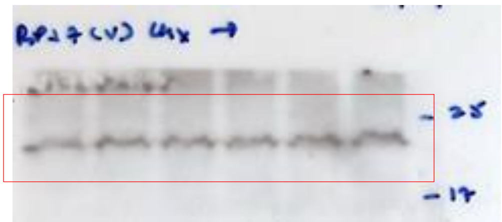

Fig. 5G- RPS7 (upper panel)

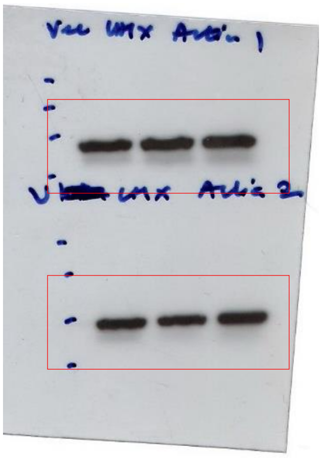

Fig. 5G - Actin (upper panel)

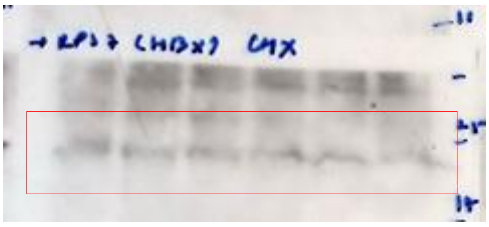

Fig. 5G - RPS7 (lower panel)

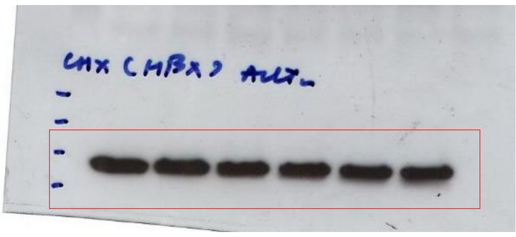

Fig. 5G - Actin (lower panel)

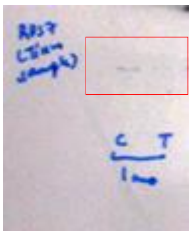

Fig. 5E - RPS7

FIG. 7

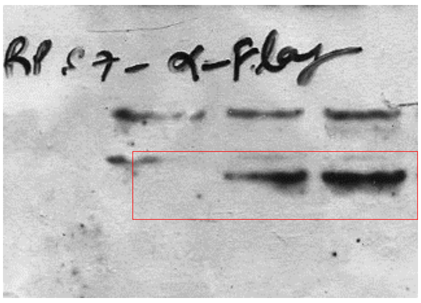

Fig. 7 Flag-RPS7
