# Supplementary material for: Stabilization of SIRT7 deacetylase by viral oncoprotein HBx leads to inhibition of growth restrictive RPS7 gene and facilitates cellular transformation
Source: Sci Rep. 2015 Oct 7;5:14806. doi: 10.1038/srep14806 (PMC4595800; doi:10.1038/srep14806)
Supplement: Supplemental data [file srep14806-s2.pdf]

Stabilization of SIRT7 deacetylase by viral oncoprotein HBx leads to inhibition of growth restrictive *RPS7* gene and facilitates cellular transformation

Vijaya Pandey and Vijay Kumar

Supplementary Table and Figures

Supplementary Table S1. Sequences of primers used

**q-PCR primers**	**Primer sequence**
SIRT7	5'- AATACTTGGTCGTCTACAC- 3'
	5'- TGCACCAGCTTCTGCTCATGC-3'
RPS7	5'-TCGCGAGATTTGGGTCTCT-3'
	5'-GGCGCTCGAACTGAACAT-3'
Actin	5'-ACCAACTGGGACGACATGGAGAAA-3'
	5'-TAGCACAGCCTGGATAGCAACGTA-3'
**ChIP primers**	**Primer sequence**
RPS7 promoter	5'-CGGCTGAAAGTAACTCTTGCAT-3'
	5'-GCTGTGGACAGGGAATTTAATC-3'

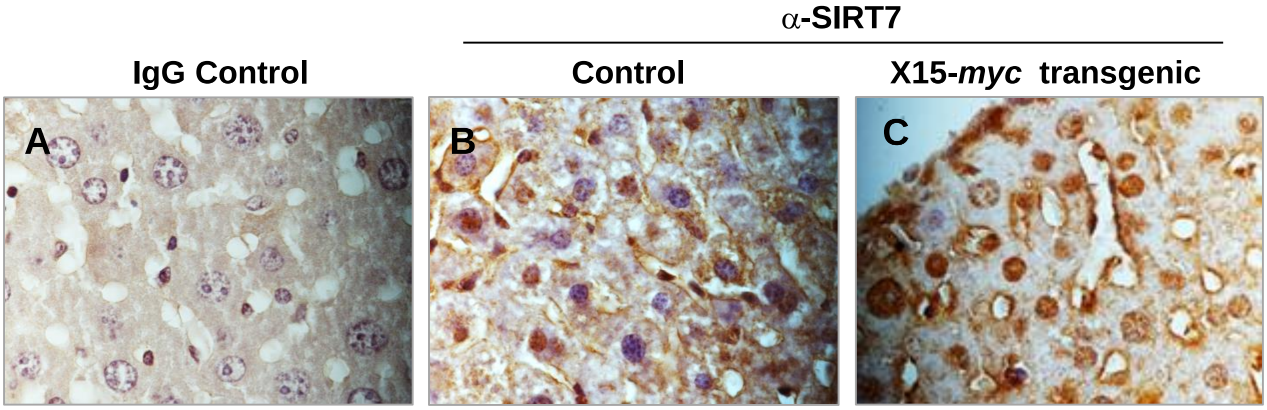

Supplementary Fig. S1. Immuno-histochemical detection of SIRT7 in X15-*myc* transgenic mouse model of HCC. Protein expression levels of SIRT7 were determined in liver sections of 5 months old X15-myc transgenic mice and healthy transgenic littermate (control) of same age group. Rabbit SIRT7 antibody was used for detection while IgG was used as negative control. IHC processing was done using Lab Vision DAB Quanto detection system. Hematoxylin was used for counterstaining. Slides were observed under Nikon Eclipse 80*i* microscope (All images, original magnification, x 600).

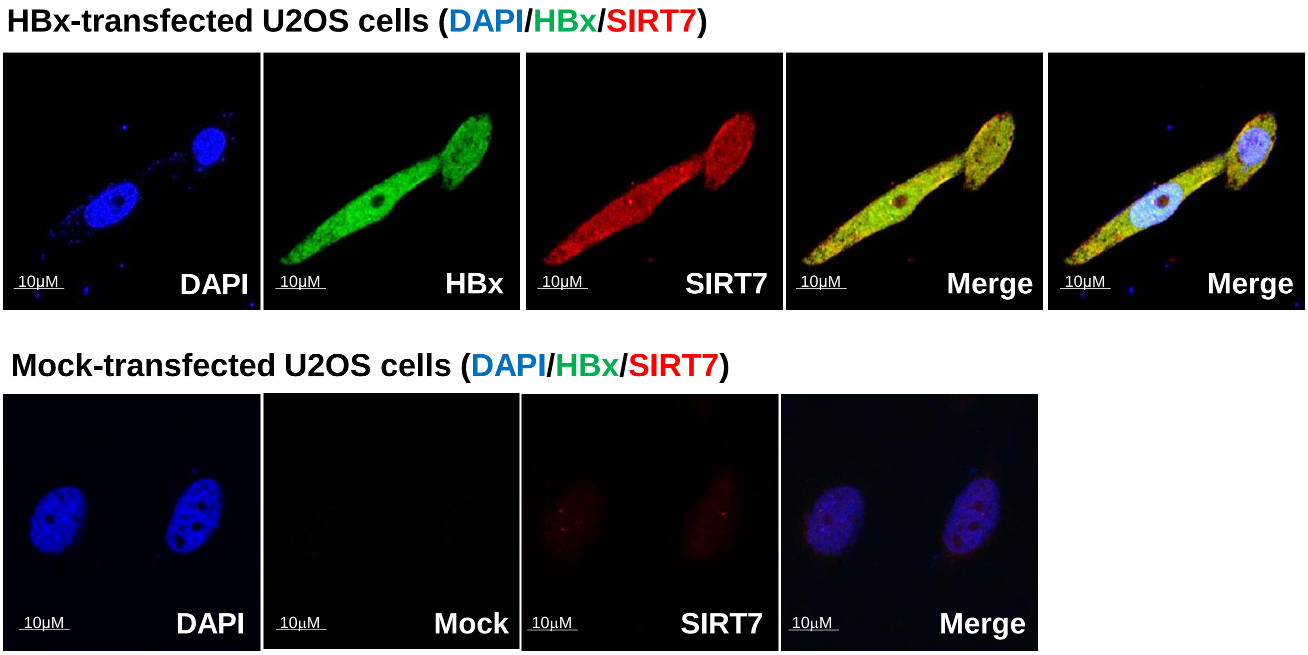

Supplementary Fig. S2. Co-localization of cellular SIRT7 and viral HBx. U2OS cells were transfected with either empty vector (mock-transfected) or HBx expression construct (HBx-transfected) for 48 h. Cell were fixed with 2% paraformaldehyde followed by immuno-staining with rabbit anti-SIRT7 and mouse anti-HBx as primary antibodies. Secondary antibodies used were Alexa594-conjugated anti-rabbit antibody for SIRT7 (red) and Alexa488-conjugated anti-mouse antibody for HBx (green). Nuclei were stained blue with DAPI. For confocal images, scale bar = 10μM.
